# Investigating the Vibrational, Magnetic and Dielectric Properties, and Antioxidant Activity of Cerium Oxide Nanoparticles

**DOI:** 10.3390/ijms232213883

**Published:** 2022-11-11

**Authors:** Nicusor Fifere, Anton Airinei, Mihai Asandulesa, Aurelian Rotaru, Elena Laura Ursu, Florica Doroftei

**Affiliations:** 1 Petru Poni Institute of Macromolecular Chemistry, 41A Grigore Ghica Voda Alley, 700487 Iasi, Romania; 2 Faculty of Electrical Engineering and Computer Science & MANSiD Research Center, Stefan cel Mare University, 13 Str. Universitatii, 720229 Suceava, Romania

**Keywords:** cerium oxide nanoparticles, raman spectroscopy magnetic properties, dielectric properties, Nyquist plots, antioxidant activity

## Abstract

Dielectric, magnetic and Raman measurements of cerium oxide nanoparticles obtained by the precipitation method are discussed. Morphological study was performed by scanning electron microscopy, confirming the formation of nanoparticles of 5–27 nm. The Raman spectra exhibited a strong band around 465 cm^−1^, corresponding to the symmetrical stretching mode of the Ce-O8 vibrational unit. The nature of the room temperature ferromagnetism of cerium oxide nanoparticles was analyzed, taking into account the oxygen defects at the surface or interface of the nanoparticles. The evolution of dielectric constant, ε′, and dielectric loss, ε″ was studied as a function of frequency at different temperatures. Additionally, the variation of the electric conductivity versus temperature was investigated. Finally, complex impedance study of the cerium oxide nanoparticles was performed.

## 1. Introduction

Nowadays, cerium oxide is among the most promising transition metal oxides for various applications as a nanomaterial, namely photocatalysis, gas sensors, solar cells, fuel cells, polishing materials, automotive catalytic converters, corrosion protection coatings for metal and alloys, and ultraviolet light detectors [1,2,3,4,5,6,7]. This wide range of applications is based on the unique properties of cerium oxide, such as high chemical stability, low toxicity, high oxygen storage ability, high transparency in visible range, low ionization potential, good redox performance, good lattice compatibility with Si, ease and cost-effective preparation, and others [1,5,8,9,10].

Cerium oxide exhibits a stable cubic fluorite (Fm3m space group) crystal structure with a lattice constant value of 5.4113 Å over a wide temperature range, from room temperature to its melting point, in which each cerium site is coordinated by eight oxygen anions and each oxygen site is coordinated by the four nearest cerium ions. This arrangement provides eight-fold coordination of cerium and four-fold tetrahedral coordination of oxygen ions [5,7,11]. This structure can be maintained at elevated temperatures. Cerium oxide is a wide bandgap semiconductor (E_g_ = 3.15–3.19 eV) at room temperature. It is particularly active in the UV range and has large exciton binding energy due to its unfilled 4f electronic configuration. The value of the bandgap energy depends on the preparation procedure and the presence of dopants and defects [1,4,12]. The distinctive characteristics of cerium oxide nanoparticles are due to the Ce^3+^/Ce^4+^ valence states and important deviations in stoichiometry. The conversion between the Ce^3+^ and Ce^+4^ oxidation states can determine the formation of high amount of oxygen defects in the lattice. The prevalence of oxygen defects increases as the nanoparticle size decreases, with their number being in direct relationship with the Ce^3+^ ion concentration [1,13,14]. To understand the effect of oxygen vacancies on the physico-chemical characteristics of cerium oxide nanoparticles, more systematic studies need to be undertaken. The strong redox ability of the Ce^3+^ and Ce^4+^ oxidation states and the presence of oxygen vacancies can determine the enhancement of the properties of cerium oxide-based materials and their use in a large range of industrial, environmental or biomedical applications. Additionally, the oxygen vacancies bring about oxygen storage capacity, catalytic activity and spintronic properties in cerium oxide nanomaterials.

Oxygen vacancies play an important role in generating magnetic properties at room temperature in cerium oxide nanostructures by changing the band structure [7,15,16]. The ferromagnetism in cerium oxide nanoparticles can be caused by the exchange interactions between impaired spins resulting from oxygen vacancies on the nanoparticle surface [17,18,19]. Additionally, the unpaired electrons in the 4f level of Ce^3+^ can be the source of ferromagnetism, but the Ce^3+^ distribution and the Ce^3^+/Ce^+4^ ratio influence the magnetization of cerium oxide nanoparticles [7,16,20]. The nature of the oxygen vacancies, their distribution in the lattice, the mixed valence of the cerium ions and the shape and size of the nanoparticles must be systematically investigated in analyses of the magnetism of cerium oxide nanoparticles, as the appearance of magnetism remains controversial.

Cerium oxide can be considered a promising candidate to replace conventional silicon oxide gate dielectrics in metal oxide semiconductors and memory devices due to its high dielectric constant, high refractive index and high thermal stability with low leakage current [21,22,23]. In addition, the cubic fluorite structure of the cerium oxide is close to that of the silicon, allowing cerium oxide-based devices to be integrated in silicon-based electronic circuits [24,25]. The oxygen vacancies and the variation of the oxidation state of Ce^3+^/Ce^4+^ couples improve the capacitive behavior and electric conductivity of nanomaterials [24,26]. The formation of these defects might significantly influence the morphology, surface and interface structure, which will affect the optical and electrical properties of cerium oxide nanostructures. Dielectric measurements are necessary to understand the conduction mechanism in these nanomaterials and the effect of structural defects on these characteristics, because, in this way, new information about the localized charge carrier behavior and ionic conductivity can be obtained [27]. Dielectric investigations and conductivity determinations are very important for the fabrication of some devices based on cerium oxide nanostructures [27]. Although systematic studies on the dielectric properties of the metal doped cerium oxide nanostructures have been performed, the dielectric properties of cerium oxide nanoparticles have not been reported to date [22,25,26,28,29].

Most of the properties of cerium oxide nanoparticles, as well as their antioxidant characteristics, are derived from Ce^3+^/Ce^4+^ redox transformations. The antioxidant activity of cerium oxide nanoparticles can be determined, to a certain extent, by the exposed surface [30]. This is because cerium oxide nanoparticles exhibit antioxidant activity through redox reactions between the oxidizing agent and the redox couple Ce^3+^/Ce^4+^ from the surface of the nanoparticles [31,32]. Previous studies have shown that Ce^3+^ from surfaces can be considered as active sites which can easily be oxidized by reactive oxygen species [33,34]. On the other hand, Lu et al. [35] demonstrated that the antioxidant properties of cerium oxide nanoparticles depend on the concentration of Ce^3+^ on the surface, albeit in different ways. The reducing character of Ce^3+^ ions on the nanoparticle surface, i.e., essentially its antioxidant activity, increases with an increase of the ceric ion concentration up to a certain limit. Above this limit, the nanoparticles show the opposite characteristic, namely, they become pro-oxidant. The thermodynamics and kinetics of such redox reactions, in solid crystalline state, depend on the structure and characteristics of the crystal lattice of the nanoparticles [31,36,37]. Under oxidizing agents, like free radicals, Ce^3+^ can shift to Ce^4+^, accompanied by oxygen binding or release [38]. These processes are determined by the concentration and type of oxygen vacancies in the crystal lattice [39]. Gunawan et al., demonstrated the importance of oxygen vacancies in the antioxidant properties of nanoparticles. They took into account the surface/bulk accessibility of the oxygen vacancies and the important role that superficial oxygen vacancies play [40]. The conclusions of such studies can be generalized, highlighting the implications of oxygen deficiency in nanoparticles in terms of capturing and reducing free radicals, even in other types of nanoparticles [41]. All these characteristics influence the physical properties of cerium oxide-based nanoparticles, such as the magnetic or dielectric properties, with an indirect impact on their surface oxide-reducing properties, thereby playing a crucial role in scavenging reactive oxygen species (ROS).

In our previous paper, we successfully obtained cerium oxide nanoparticles by the precipitation method using glycerol as a dispersion medium for cerium sulphate and with modified synthesis parameters (temperature, precipitating agent) [42]. The prepared nanostructures were characterized by X-ray diffraction, diffuse reflectance spectra, emission spectra, XPS, and transmission electron microscopy. These cerium oxide nanoparticles exhibited excellent antimicrobial activity against pathogens such as Escherichia coli and Staphylococcus aureus. The current study investigates the Raman spectra, scanning electron microscopy data, and magnetic and dielectric behavior of cerium oxide nanoparticles. Additionally, the antioxidant activity of the cerium oxide nanoparticles, using DPPH as a radical compound, is discussed.

## 2. Results and Discussion

### 2.1. Raman Spectra

**Figure 1:**
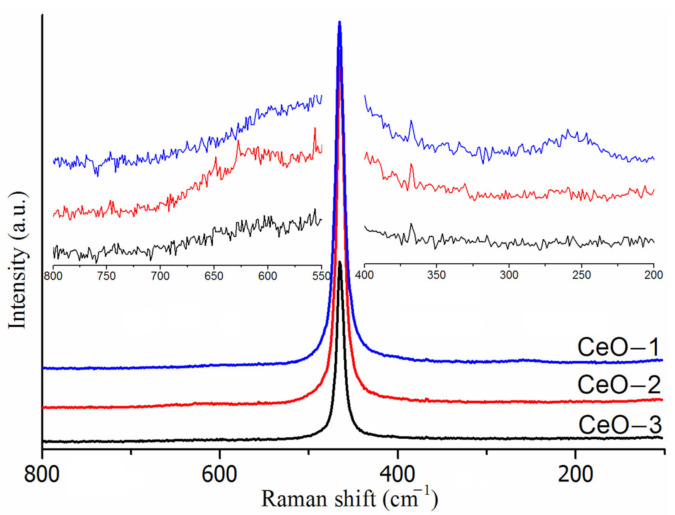
Raman spectra of cerium oxide nanoparticles.

Raman spectra give information about the purity, crystalline nature and defect levels of nanomaterials. Figure 1 illustrates representative Raman spectra of cerium oxide nanoparticles. As shown an intense Raman band at around 465 cm^−1^ was observed for all the cerium oxide samples. This band was assigned to the symmetrical stretching mode of the Ce-08 vibrational units with fluorite type structures (F_2g_ vibration of CeO_2_) [43,44,45,46], consistent with the XRD data. In addition, two Raman bands of low intensity located around 260 cm^−1^ and 595 cm^−1^ can occur on the high and low energy sides of the main Raman mode. These additional bands can be assigned to doubly degenerate transverse optical (2TA) mode in the cerium oxide lattice and to the non-degenerate longitudinal optical mode (LO) related to the defects generated by oxygen vacancies after the conversion of Ce^4+^ to Ce^3+^ ions [47,48,49,50]. These Raman modes have low intensities for CeO-2 and CeO-3 samples, while CeO-1 showed a well-defined mode at 260 cm^−1^ (Figure 1). Notably, the Raman mode at 595 cm^−1^ for sample CeO-1 exhibited a higher intensity compared to the other samples, indicating a high amount of oxygen vacancies in that sample. Relating to the position of the Raman active fundamental mode of bulk cerium oxide (464 cm^−1^) [51], the cerium oxide nanoparticles presented small shifts to longer wavenumbers. The CeO-1 sample exhibited a higher full width at half maxima due to the smaller crystallite size as compared to CeO-2 and CeO-3 (Table 1).

According to the Raman line broadening, the particle size can be calculated from the dependence of the half-width (*Γ*) on the inverse of crystal size (*D_R_*) using Equation (1) [52].

(1) $$\Gamma \left({\mathrm{cm}}^{-1}\right)=5.48+98.4/D_{R\;}\left(\mathrm{nm}\right)$$

Using Equation (1), the size of CeO_2_ nanoparticles was found to be to 15.97 nm (CeO-1), 21.40 nm (CeO-2) and 25.67 nm (CeO-3), respectively. These values are in accordance with the results obtained from X-ray diffractograms, except for the CeO-3 sample, where the calculated particle size based on Raman spectra was higher, probably due to the anisotropic nature of the nanoparticles.

The concentration of the oxygen vacancies can be evaluated based on the spatial correlation model using the relation between the correlation length and the grain size [15,53,54]:

(2) $$L\;\left(\mathrm{nm}\right)={\left\{{\left[\alpha /\left(2D_{R}\right)\right]}^{2}\cdot \left[{\left(D_{R}-2\alpha \right)}^{3}+4D_{R}^{2}\alpha \right]\right\}}^{1/3}$$

where *L* represents the correlation length, i.e., the average distance between two lattice defects and α is the radius of cerium oxide molecule determined to be 0.34 nm [53]. The defect concentration *N* (cm^−3^) may be related to the correlation length, using the following equation [44,54]:

(3) $$N=\frac{3}{4\pi L^{3}}$$

The defect concentration is given in Table 1 for cerium oxide nanoparticles. As shown, sample CeO-1 had the highest content of oxygen vacancies on its surface, confirming the fact that small grain sizes induce higher defect concentrations.

Looking at the Raman spectra of cerium oxide nanoparticles, one can see that an increase in the grain size is accompanied by a decrease in the F_2g_ vibration wavenumber and a slight decrease in the half-width of the same Raman band. As mentioned, lattice defects are expected to be the reason for the changes in the position and widening of the F_2g_ vibrations [44,47,51]. The low intensity of the Raman band around 595 cm^−1^ and the presence of Ce^3+^ in the XPS data indicated that the disorder in the oxygen sublattice of the nanoparticles was reduced and the adsorbed oxygen species on the nanoparticle surface could contribute to a greater extent to these parameters [47,51].

### 2.2. Morphology and Elemental Composition of Cerium Oxide Nanoparticles

**Figure 2:**
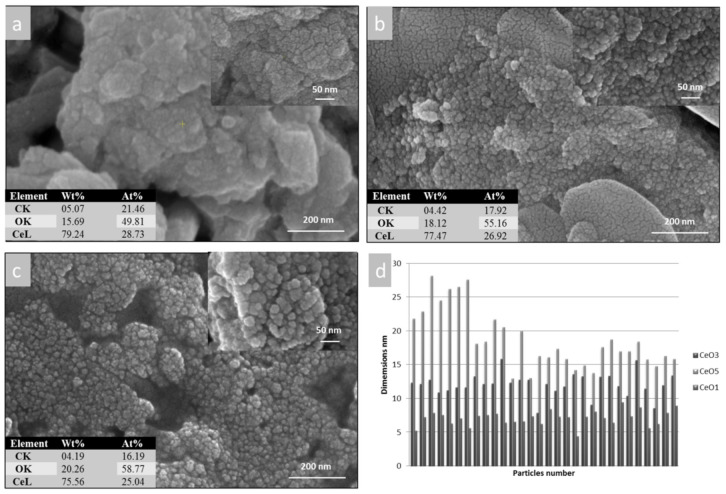
Morphology with elemental composition of synthetized nanoparticles: (**a**) CeO-1, (**b**) CeO-2, (**c**) CeO-3, (**d**) histogram with the particles size, as measured by Image j software.

Figure 2 shows SEM micrographs of the three cerium oxide samples and (as inset tables) their elemental composition. In addition to the SEM images, Figure 2d shows a histogram with the particle size distribution for the samples under study. In Figure 2a, it can be seen that the cerium oxide nanoparticles had mainly spherical like morphologies with a tendency to form agglomerations. Particle sizes were determined from SEM images using Image J Software. Thirty particles with a well-defined spherical shape were measured and then a histogram was created. The sizes of the CeO-1 nanoparticles were between 4 and 9 nm.

The morphology of the CeO-2 sample is presented in Figure 2b. In this case, an increase in the size of the cerium oxide nanoparticles and a decrease in the agglomeration tendency can be observed. Here, the spherical morphology of the nanoparticles was much more evident. The nanoparticle sizes were between 9 and 15 nm.

The CeO-3 sample also exhibited spherical nanoparticles with larger dimensions (12–27 nm) which presented as individual particles Figure 2c. It may be observed that depending on the synthesis method, nanoparticles with different dimensions and with a different agglomeration tendency can be obtained. The composition of the samples was confirmed by EDX. The insert tables alongside the SEM micrographs show the presence of Ce and O. In addition to these elements, there was also C, which can be attributed to the carbon tape on which the samples were fixed. EDX analysis is a qualitative analysis method that confirms the elemental composition of nanoparticles.

### 2.3. Magnetic Properties

**Figure 3:**
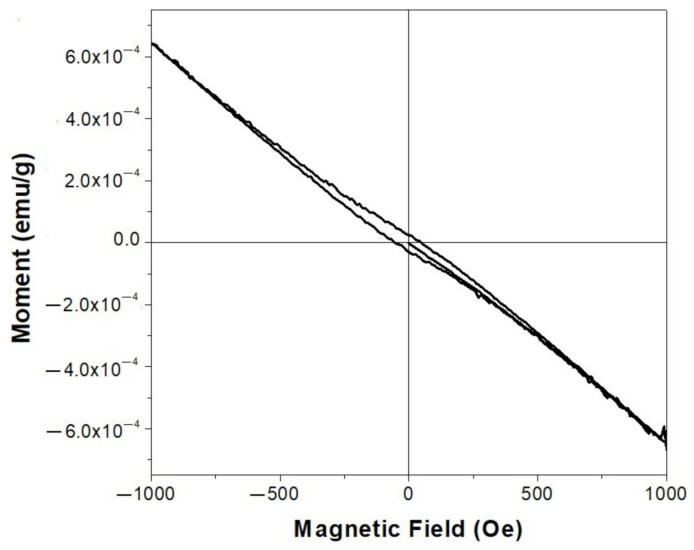
M-H hysteresis loops for sample CeO-1.

The magnetic properties of the as-synthetized cerium oxide nanoparticles were examined using a vibrating sample magnetometer at room temperature. The magnetic hysteresis (M-H) loops of the CeO-1 sample are displayed in Figure 3. It can be observed that this sample exhibited weak ferromagnetism at room temperature. Some magnetic parameters, namely saturation magnetization, remanent magnetization (Mr) and coercivity (He), were estimated from the M-H curve to have the following values: 8.0 × 10^−5^ emu/g, 2.8 × 10^−5^ emu/g and 116.9 Oe, respectively. These values are comparable to those of some earlier reported data for pure cerium oxide or doped cerium oxide nanoparticles [11,43,55,56]. From our XPS analysis, the development of oxygen vacancies on the nanostructure surface and the presence of Ce^3+^ ions were confirmed [42]. In this way, the ferromagnetic behavior of the CeO-1 sample may have been the result of interactions between oxygen vacancies and Ce^3+^ ions which determine the alignment of spins in the local environment. However, this relationship between the presence of oxygen deficiencies and ferromagnetism requires further investigations.

**Figure 4:**
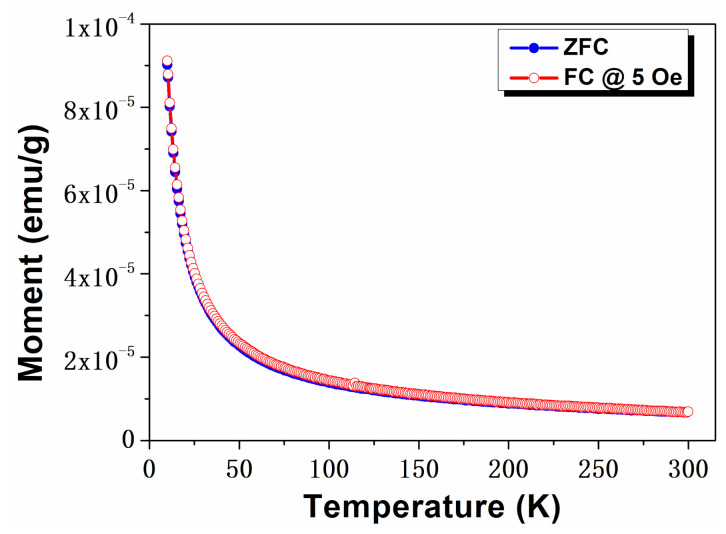
ZFC and FC M−T plots for CeO-2 sample at field of 500 Oe.

In addition to field dependent magnetic analysis, the temperature dependence of the magnetization of the CeO-2 sample was analyzed in zero field cooling (ZFC) and field-cooling modes in order to explore the observed magnetism. The combined ZFC-FC curves are displayed in Figure 4. The M-T plot for CeO-2 nanoparticles showed that the FC and ZFC curves exhibited similar behavior and that the field applied below and above the coercitive field did not modify the ZFC and FC traces. The bifurcation between the ZFC and FC curves suggest the presence of ferromagnetic ordering in the heat-treated sample, accompanied by an increase of coercive field [14,57,58]. Initially, the magnetization was not dependent on the temperature in both ZFC and FC plots, whereas when the temperature decreased, a sharp increase of magnetization was observed (Figure 4). Although the ZFC and FC curves seem to overlap, a zoomed in representation of these curves shows a slight separation, which confirms the existence of ordering in the sample. Additionally, the significant increase of magnetization may have been due to the presence of a magnetic ordering in the CeO-2 sample [59].

**Figure 5:**
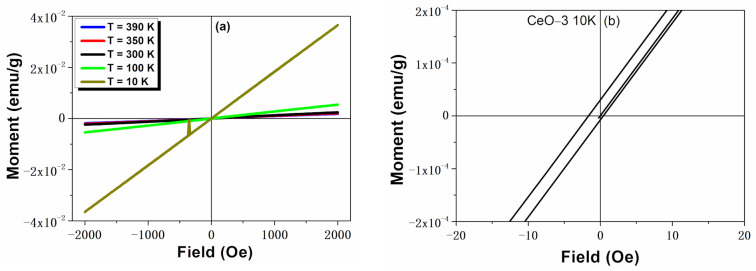
Magnetization curves of CeO-2 (**a**) at different temperatures, (**b**) in the low field region at 10 K.

The magnetization curves of the CeO-2 sample recorded at different temperatures (10, 100, 300, 390 K) are illustrated in Figure 5a. For the measured temperatures, the same quantitative results were obtained and the magnetization increased very rapidly with a lowering of the temperature. The M-H curves show the hysteretic nature with a paramagnetic contribution, as evidenced in Figure 5b. Saturation magnetization was not observed, even under an applied magnetic field of 2000 Oe. The coercivity (Hc) values for CeO-2 were 7.7 Oe at 100 K and 14.9 Oe at 300 K. These values much lower than those for the CeO-1 sample (116.9 Oe, 300 K).

In this case, the results suggest the existence of a mixed phase of ferromagnetic and paramagnetic in the CeO-2 sample, in agreement with previous papers [60,61]. It was demonstrated that the presence of surface oxygen deficiency determines the magnetic ordering within cerium oxide nanostructures [11,13,18,62,63]. The XPS data indicated a higher content of oxygen vacancies in CeO-1, which exhibited a dominant ferromagnetic response, whereas in the CeO-2 sample having a higher content of adsorbed oxygen the ferromagnetism was diminished [42].

### 2.4. Dielectric Behavior

**Figure 6:**
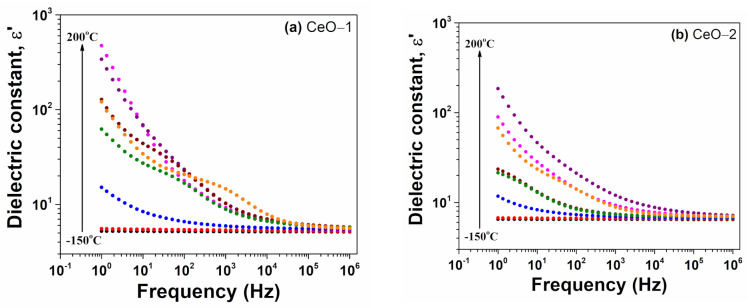
Variation of the dielectric constant as a function on frequency at different temperatures of cerium oxide nanoparticles: (**a**) CeO-1, (**b**) CeO-2.

The applied electrical field frequency response of the dielectric constant (ε′) for cerium oxide nanoparticles at selected temperatures is shown in Figure 6 over a frequency range of 1 Hz–10^6^ Hz. As a general trend, the dielectric constant decreased rapidly with the applied frequency, reaching a plateau domain due to the low capacity of the induced dipoles to follow the oscillations of the external electrical field at high frequencies. On the other hand, as the temperature increased, the value of ε′ was heightened over entire frequency range. These results are similar with previous reported data on the dielectric behavior of the cerium oxide nanoparticles [9,23,25,64,65]. At low frequencies, the dielectric constant exhibited very high values due to the presence of space charge polarization, because the charge carriers accumulated at the grain interfaces and insulating grain boundaries, which provided a high dielectric constant [25,29,64]. The electric applied field determines the movement of the space charges at interface, leading to the creation of dipole moments along the field direction, which causes space charge polarization. At low frequencies, the induced electric moment or the electron hopping can synchronize with the applied field, yielding higher dielectric constant values. It can be noted that the inhomogeneities formed in cerium oxide nanostructures (oxygen vacancies, grain boundary defects or surface defects) can enhance the polarization degree and the ε′ value. Additionally, the dielectric properties can be influenced by changing oxidation states of cerium, i.e., between 3+ and 4+, creating different amounts of dipole moments [29,66,67,68]. In the high frequency region, the effect of space charge polarization was strongly diminished, revealing a slight decrease of ε′ with increasing frequency. As the frequency increased, the electric dipoles failed to follow the quick electric alternating field, and a constant value was obtained for dielectric constant. Similar frequency dependences of the dielectric constant have been reported elsewhere [9,25,64]. At low frequencies, as the temperature increased, the dielectric constant increased, and at high frequencies, ε′ remained practically at a constant value (Figure 6) due to the conductivity enhancement and higher charge carrier mobility [10].

**Figure 7:**
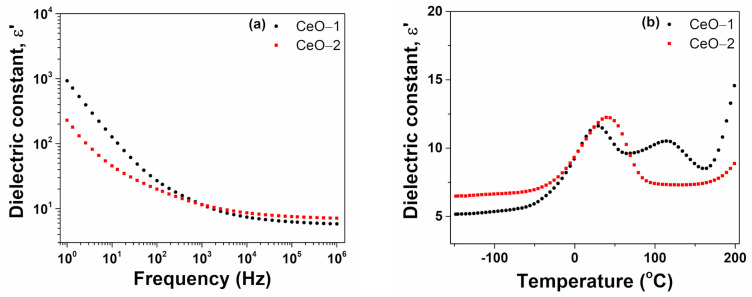
Dielectric constant dependence of cerium oxide nanoparticles (**a**) on the frequency at room temperature, (**b**) on temperature at 1 kHz.

Considering the ε′ (f) spectra of cerium oxide nanoparticles selected at room temperature, we noticed that the effect of the space charge polarization was more pronounced for the CeO-1 sample, while the magnitude of ε′ was comparable for both samples, suggesting a similar dipolar activity (Figure 7a). For example, at a frequency of 1Hz, the value of ε′ was found to be 926 for CeO-1 and 229 for CeO-2, while at 1 MHz, the value of ε′ was 11.5 for both samples. The variation of the dielectric constant as a function of temperature for cerium oxide nanoparticles is shown in Figure 7b at a frequency of 1 kHz. The CO-2 sample presented a dielectric band centered around 40 °C, while the CeO-1 sample revealed two bands, one around 30 °C and a supplementary one at 115 °C. Such peaking behavior of the dielectric constant has been reported in few cases in the literature for cerium oxide nanostructures and revealed the presence of a dielectric relaxation mechanism [66,68]. However, this behavior was not noticed for other cerium oxide nanostructures [23]. As shown in Figure 7b, there was a transition phase for CeO-2, which had a higher grain size toward higher temperatures than CeO-1. This dielectric relaxation can be determined by the defect-dipole polarization effect [66].

**Figure 8:**
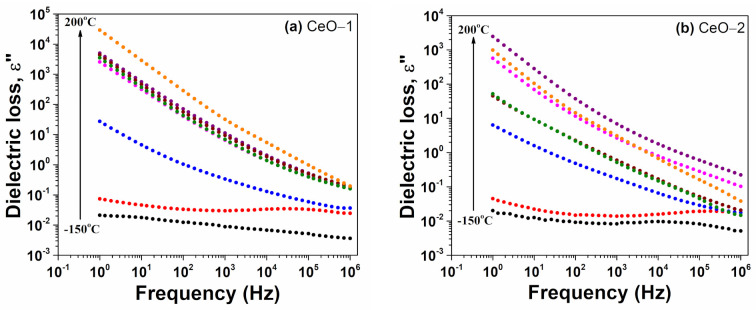
Dielectric loss dependence on frequency at various temperatures for cerium oxide nanoparticles: (**a**) CeO-1, (**b**) CeO-2.

The frequency dependent dielectric loss for cerium oxide nanoparticles, in a frequency window between 1 Hz and 1 MHz, is shown in Figure 8. The evolution of the dielectric loss based on frequency was found to have almost the same trend as that of dielectric constant. At low temperatures, i.e., between −150 °C and −100 °C, small values of ε′ were recorded, i.e., even lower than 0.1 in the entire frequency range. At higher temperatures, the values of dielectric loss decreased with an increase in frequency. The high values of dielectric loss at low frequencies may have been due to the charge lattice defect of the space charge polarization, because the electric dipoles can follow the variations of the applied electric field, and the contribution of the space charge polarization becomes greater [29,67]. This observation is in accordance with that detected on the isothermal plots of ε′ as a function of frequency. At temperatures between 0 °C and 100 °C, the ε″ (f) dependencies, obtained in the double logarithmic plot, exhibited a linear decreasing tendency, with a slope close to −1. This regime is characteristic of the presence of mobile charge carriers [69]. Figure 8 shows that a linear decreasing tendency of ε″ appeared at lower temperatures for CeO-1 than for CeO-2. Additionally, for CeO-2, which had a larger crystallite size, the grain boundaries per volume unit and the defects in the vicinity of the grain boundaries were diminished, leading to lower dielectric loss.

**Figure 9:**
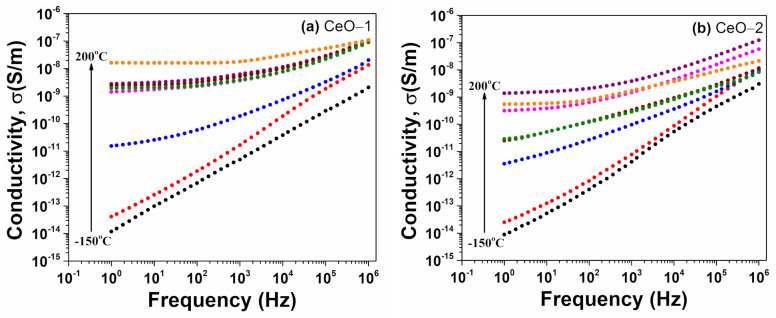
Variation of conductivity versus frequency at various temperatures for cerium oxide nanoparticles (**a**) CeO-1, (**b**) CeO-2.

Following the behavior of the conductivity as a function of frequency (Figure 9), two different frequency domains can be observed: a linear increase in conductivity with frequency, attributed to the dipolar relaxation processes of the materials, and a frequency independent plateau which was specific to the dc conductivity. The plateau region occurred at low frequencies and high temperatures. This correlates well with sharp drop of ε′ and the linear decrease of ε″ with increasing frequency.

The contribution of the grain, grain boundaries and the electrode can be depicted using complex impedance analysis. In this way, for the accurate determination of the dc-like conduction in cerium oxide nanoparticles, typical Nyquist plots were obtained, representing the real impedance part *Z*′ versus the modulus of the imaginary part of impedance *Z*″. The impedance *Z*″ against *Z*′ plots were obtained using the well-known Cole equation:

(4) $$Z^{*}\left(\omega \right)=Z^{\prime }\left(\omega \right)+Z^{\prime \prime }\left(\omega \right)=\frac{R_{dc}}{1+{\left(i\omega \tau \right)}^{\alpha }}$$

**Figure 10:**
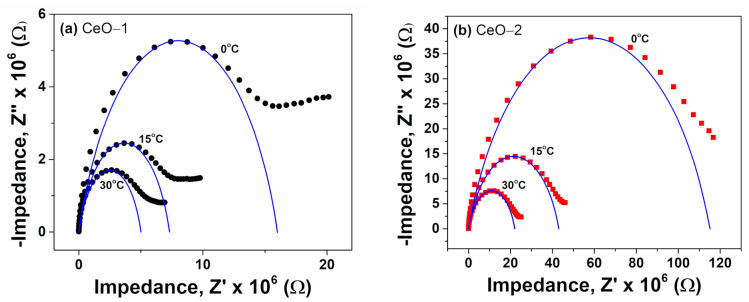
Experimental dielectric data and simulated Cole-Cole plots at various temperatures for (**a**) CeO-1 and (**b**) CeO-2.

In the Equation (4), the complex impedance of the material, Z*(*ω*) is related to the ohmic resistance, *R_dc_*, the angular frequency, *ω*, the characteristic time constant, *τ*, and an empirical exponent, *α* [70,71]. Typical fits based on the Cole equation were processed with the WinFit software provided by Novocontrol. Figure 10 depicts the Cole-Cole plots (Nyquist plots) corresponding to different temperatures for cerium oxide nanoparticles. The impedance spectra are characterized by semicircle arcs whose area decreases as the temperature increases. These semicircles are deformed in shape, suggesting that the grain and grain boundary region contributions are overlapped to form a single semicircle [72]. The decrease of the radius of the *Z′*-*Z″* semicircular arcs with increase in temperature indicated an increase in conductivity and the corresponding decrease of the grain and grain bounding resistance, as expected in electronic semiconductors. As shown in Figure 10, the sample CeO-2 exhibited a higher impedance value as compared to CeO-1, probably due to the greater crystallite size of the latter. Other studies have found that the Nyquist plot radius increases as the temperature increases [29,73], confirming the temperature dependence of the electrical behavior of cerium oxide nanoparticles. From the simulated Nyquist plots, the ohmic resistance can be obtained from the intercept of the semicircle with *Z′* axis. In this way, the dc conductivity, *σ_dc_*, was retrieved with the following equation:

(5) $$\sigma_{dc}=\frac{1}{R_{dc}}\cdot \frac{d}{A}$$

where *d* and *A* represent the thickness and surface area of the sample.

**Figure 11:**
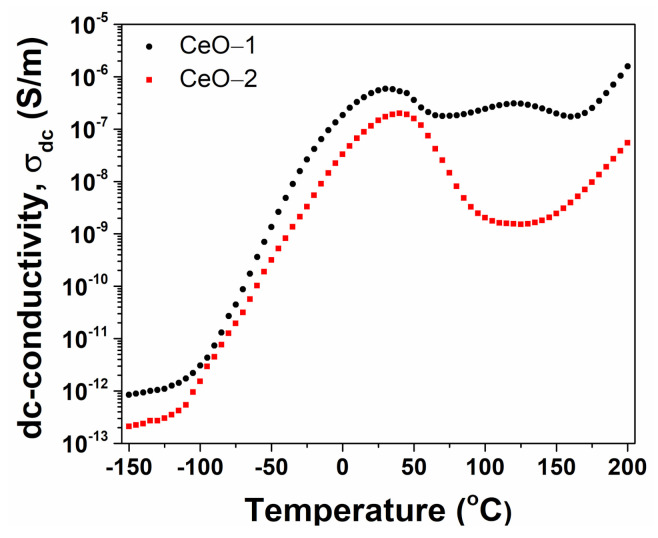
Conductivity dependence on the temperature estimated from the Cole-Cole plots for CeO-1 and CeO-2 samples.

The evolution of the dc conductivity against temperature for nanoparticle samples is represented in Figure 11. Selected numerical values of dc conductivity at different temperatures are reported in Table 2. At temperatures between −150 °C and 35 °C, both samples revealed similar behavior, with a gradual increase of conductivity as the temperature increased, suggesting the semiconducting behavior of the material. For both samples, an inflection point was observed at around 35 °C. With further increase of temperature, a supplementary band was found on the temperature dependence of *σ_dc_* for CeO-1. The conductivity of CeO-1 was noticeably higher than that of CeO-2 in the entire temperature range, as expected for cerium oxide nanoparticles with lower dimensions and in agreement with Raman spectral data.

**Figure 12:**
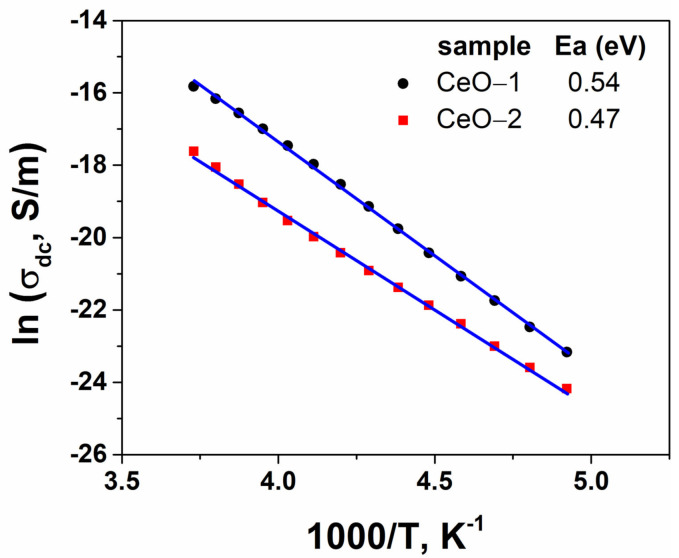
Temperature dependence of conductivity for cerium oxide nanoparticles.

The continuous increase of *σ_dc_* from −70 °C to −5 °C may have been related to the long-range movement of charge carriers through the cerium oxide lattice when an external alternating electric field was applied. The variation of the conductivity against temperature can be adequately processed by the Arrhenius Equation (6):

(6) $$\sigma =\sigma_{0}\cdot exp\left(-\frac{E_{a}}{kT}\right)$$

where *σ_o_* is a pre-exponential factor, *E_a_* represents the activation energy of the dc conductivity, *k* is the Boltzmann constant and *T* denotes the absolute temperature. Figure 12 illustrates the linear dependence of the electrical conductivity as a function of the reciprocal temperature for the cerium oxide nanoparticles. The slopes estimated from the plots are directly related to the activation energy of the sample. The charge carriers of CeO-1 had an activation energy of 0.54 eV, while for CeO-2, a lower value was found (0.47 eV) (Table 2). The obtained activation energy values for the conductivity were lower than previous reported data [9,29,74]. Based on these investigational data, the present cerium oxide nanostructures can be regarded as possible useful materials for manufacturing capacitors and for use in devices operating at high frequencies.

### 2.5. Antioxidant Activity

**Figure 13:**
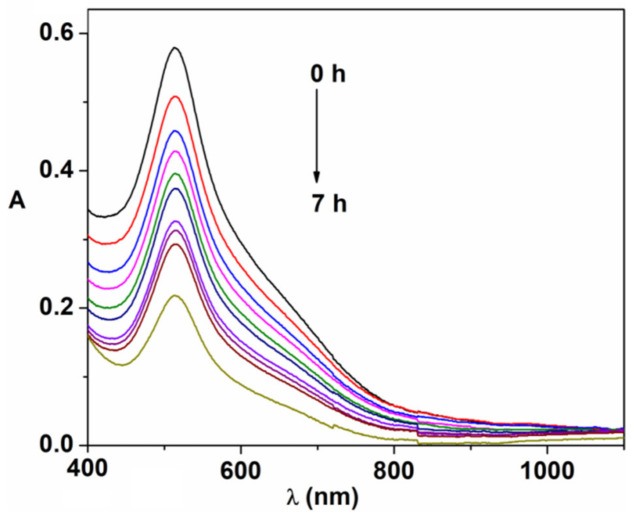
UV−Vis response of DPPH as a function of contact time with cerium oxide nanoparticles.

The antioxidant properties of CeO-1 and CeO-3 were investigated by the free radical method using DPPH·(1,1-diphenyl-2-picrylhydrazyl) as an oxidizing agent. The reaction of DPPH· with cerium oxide nanoparticles consists of the reduction of free radicals by electron donating partners, which can be cerium oxide nanoparticles, via a Ce^3+^/Ce^4+^ redox couple [35]. The antioxidant activity of the cerium oxide nanoparticles was tested spectophotometrically by following the decrease in intensity of the absorption band of DPPH at 515 nm. This degradation process resulted in the discoloration of the DPPH solution from purple to pale yellow (Figure 13). The antioxidant activity or inhibition rate was calculated as follows [75]:

(7) $$AA\%=\left(1-\frac{A_{sample}-A_{blanck}}{A_{ref}}\right)\cdot 100$$

where *A* is the absorbance at 515 nm, with particular subscripts referring to the sample (*A_sample_*), consisting of DPPH solution with nanoparticle suspension, blank solution (*A_blank_*) with nanoparticle suspension at the same concentration and reference (*A_ref_*), consisting of DPPH solution at the same concentration but without nanoparticles.

**Figure 14:**
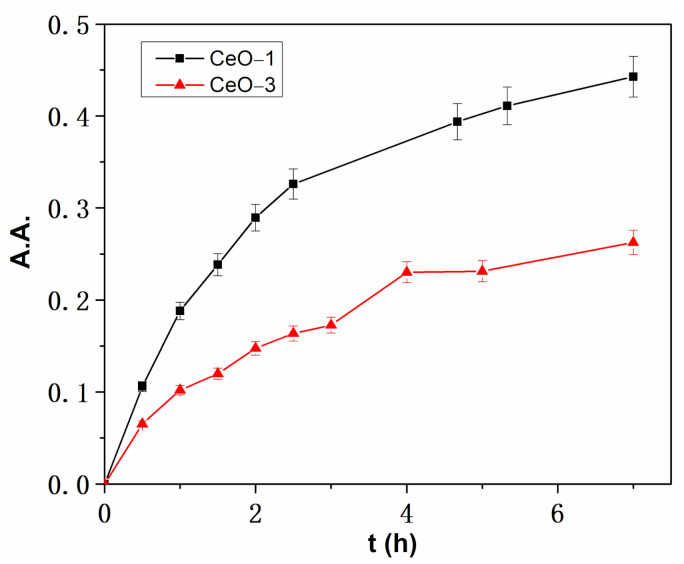
Evolution of the DPPH absorbance over time in the presence of cerium oxide nanoparticles.

The results shown in Figure 14 demonstrate that both samples exhibited a scavenging effect against DPPH free radicals. This was determined by the presence in nanoparticles of Ce^3+^ ions, which are capable of oxidizing to Ce^4+^, yielding an electron to the free radical of DPPH, i.e., reducing it [76]. Basically, the scavenging ability of the cerium oxide nanoparticles was enhanced by the ability of Ce^3+^ ions to oxidize in the crystal lattice of cerium oxide nanoparticles. From our data, it was apparent that CeO-1 presented a higher scavenging effect than CeO-3, which means that in the crystal structure of the former, Ce^3+^ ion oxidation was favored more than for CeO-3. In this respect, it is necessary for the Ce^3+^ ions to generate active oxygen vacancies in the crystal lattice of the nanoparticles. The oxygen deficiency sites in nanoparticles act as electron traps for free radicals, facilitating the transfer of electrons from Ce^3+^ [35]. These results are correlated with the magnetic measurements in which a dominant ferromagnetic response was found due to the oxygen deficiency in the nanocrystals of the CeO-1 sample, which increased its scavenging ability. In the case of CeO-3, the mixed ferromagnetic and paramagnetic phase indicated that the presence of the adsorbed oxygen decreased the oxygen deficiency and lowered the antioxidant properties. XPS studies also proved that even though CeO-1 had a lower content of Ce^3+^ than CeO-3, the former was oxygen deficient while the latter was rich in oxygen, i.e., with an oxygen content higher than stoichiometric value [42]. The oxygen deficient sites in CeO-1 were the active sites that increased the possibility of Ce^3+^ oxidation, thereby also increasing the scavenging ability of the sample as compared to CeO-3.

## 3. Materials and Methods

Cerium oxide nanoparticles were prepared by the precipitation method using cerium(IV) sulphate (>99% Sigma-Aldrich/Merck, Darmstadt, Germany) as a precursor and sodium hydroxide (>98%, Sigma-Aldrich/Merck, Darmstadt, Germany) or ammonium hydroxide (25%, Chemical Company, Iasi, Romania) as a precipitating agent (Table 1), as previously described [42,43]. Raman measurements were made on a micro-Raman system (inVia Reflex, Renishaw, New Mills, United Kingdom) with an excitation wavelength of 633 nm, provided by a 633 nm He-Ne laser at room temperature. A 50 X objective was utilized to irradiate the sample and to collect the backscattered light. Magnetic properties were investigated using a MPMS3 (7T) SQUID magnetometer (Quantum Design, San Diego, CA, USA) at room temperature. Particle morphology and the elemental composition of the synthesized samples were analyzed with a Verios G4 UC scanning electron microscope (Thermo Scientific, Brno, Czech Republic) equipped with energy dispersive X-ray spectroscopy analyzer (Octane Elect Super SDD detector, EDAX-AMETEK, Mahwah, NJ, USA). The samples were fixed on aluminum stubs with double-adhesive carbon tape and coated with 10 nm platinum using a Leica EM ACE200 Sputter coater (Leica Microsystem, Vienna, Austria) to provide electrical conductivity and to prevent charge buildup during exposure to the electron beam. SEM investigations were performed in high vacuum mode using a secondary electron detector (Everhart-Thornley detector, ETD) at an accelerating voltage of 10 kV. The average particle size was calculated using ImageJ software. Broadband dielectric spectroscopy (BDS) measurements were carried out with a Novocontrol Concept 40 broadband dielectric spectrometer (Novocontrol Technologies GmbH, Montabaur, Germany). The powder samples were compressed (room temperature, pressure about 10 ton) into pellets of 13 mm in diameter and a thickness of around 0.4 mm. Then, the pellets were placed between two gold coated plate electrodes and the amplitude of the external alternating field was set to 1 V. An alternating electrical field was applied with an Alpha-A High Performance Analyzer in a frequency window between 1 Hz and 1 MHz. The dielectric spectra were recorded isothermally in a temperature range between −150 °C and 200 °C using a Novocontrol Quatro Cryosystem device.

## 4. Conclusions

In summary, the detailed Raman characteristics and magnetic and dielectric properties of cerium oxide nanoparticles prepared by the precipitation method were reported. The Raman results revealed that the cerium oxide nanoparticles had a face centered cubic (FCC) structure. EDX analysis confirmed the stoichiometry of all constituent elements. Vibrating sample magnetometric study indicated that the cerium oxide nanoparticles were ferromagnetic in nature. The dielectric characteristics of cerium oxide nanoparticles showed that the dielectric constant and dielectric loss decreased, while the electric conductivity increased, with an increase of frequency. The decrease of the dielectric loss as the frequency increased may have been due to the space charge polarization. The dependence of the dc conductivity on the temperature obeyed Arrhenius’ law. The activation energy for conductivity was found to be around 0.50 eV for the as-prepared samples. The impedance spectra were characterized by semicircle arcs, showing the contribution of the grain boundaries to the total resistance of the samples.

## Figures and Tables

**Table 1:** Raman and XRD parameters of cerium oxide nanoparticles.

Sample	T (°C)	Precipitating Agent	D_XRD_ (nm)	ν (F_2g_) (cm^−1^)	D_R_(nm)	N (cm^−3^)
CeO-1	80	NaOH	13.62 ± 1.64	465.2	15.97 ± 1.71	5.37·10^21^
CeO-2	25	NaOH	15.48 ± 1.35	464.6	21.40 ± 1.83	2.83·10^21^
CeO-3	25	NH_4_OH	17.61 ± 1.61	464.9	25.67 ± 1.94	2.66·10^21^

**Table 2:** Numerical values of *σ_dc_* retrieved from the Cole-Cole fittings at low (−100 °C), room (25 °C) and high (200 °C) temperatures for cerium oxide nanoparticles.

Sample	*σ_dc_* (S/m)		
	−100 °C	25 °C	200 °C
CeO-1	3.1 × 10^−12^ (±0.07 × 10^−12^)	5.5 × 10^−7^ (±0.14 × 10^−7^)	1.6 × 10^−6^ (±0.04 × 10^−6^)
CeO-2	1.5 × 10^−12^ (±0.03 × 10^−12^)	1.5 × 10^−7^ (±0.03 × 10^−7^)	5.5 × 10^−8^ (±0.11 × 10^−8^)

## Data Availability

Data presented in this study are available upon reasonable request from the corresponding author.
